# Chloroethylating anticancer drug-induced mutagenesis and its repair in *Escherichia coli*

**DOI:** 10.1186/s41021-019-0123-x

**Published:** 2019-04-05

**Authors:** Yoko Yamada, Shinji Watanabe, Keinosuke Okamoto, Sakae Arimoto, Eizo Takahashi, Kazuo Negishi, Tomoe Negishi

**Affiliations:** 1 Graduate School of Medicine, Dentistry, and Pharmaceutical Sciences, Okayama University, Tsushima-naka, Kita-ku, Okayama, 700-8530 Japan; 2 Faculty of Pharmaceutical Sciences, Okayama University, Tsushima-naka, Kita-ku, Okayama, 700-8530 Japan; 3 Nihon Pharmaceutical University, Ina, Kita-Adachi-Gun, Saitama, 362-0806 Japan; 4 Present address: Collaborative Research Center of Okayama University for Infectious Diseases in India, National Institute of Cholera and Enteric Diseases JICA Building ID Hospital Campus, Beliaghata Kolkata, 700010 India

**Keywords:** Chloroethylnitrosourea, Nimustine (ACNU), Carmustine (BCNU), DNA repair, Recombination, Interstrand cross-link (ICL), *Escherichia coli*

## Abstract

**Background:**

Chloroethylnitrosourea (CENU) derivatives, such as nimustine (ACNU) and carmustine (BCNU), are employed in brain tumor chemotherapy due to their ability to cross the blood-brain barrier. They are thought to suppress tumor development through DNA chloroethylation, followed by the formation of interstrand cross-links (ICLs) that efficiently block replication and transcription. However, the alkylation of DNA and ICLs may trigger genotoxicity, leading to tumor formation as a side effect of the chemotherapeutic treatment. Although the involvement of *O*^6^-alkylguanine-DNA alkyltransferase (AGT) in repairing chloroethylated guanine (*O*^6^-chloroethylguanine) has been reported, the exact lesion responsible for the genotoxicity and the pathway responsible for repairing it remains unclear.

**Results:**

We examined the mutations induced by ACNU and BCNU using a series of *Escherichia coli* strains*,* CC101 to CC111, in which reverse mutations due to each episome from F’101 to F’106 and frameshift mutations due to each episome from F’107 to F’111 could be detected. The mutant frequency increased in *E. coli* CC102, which can detect a GC to AT mutation. To determine the pathway responsible for repairing the CENU-induced lesions, we compared the frequency of mutations induced by CENU in the wild-type strain to those in the *ada, ogt* (AGT-deficient) strain, *uvrA* (nucleotide excision repair (NER)-deficient) strain, mismatch repair (MMR)-deficient strains, and *recA* (recombination deficient) strain of *E. coli* CC102. The frequencies of mutations induced by ACNU and BCNU increased in the *ada, ogt* strain, demonstrating that *O*^6^-chloroethylguanines were formed, and that a portion was repaired by AGT.

Mutation induced by ACNU in NER-deficient strain showed a similar profile to that in AGT-deficient strain, suggesting that an NER and AGT play at the similar efficacy to protect *E. coli* from mutation induced by ACNU. *O*^6^-Chloroethylguanine is reported to form ICLs if it is not repaired. We examined the survival rates and the frequencies of mutations induced by ACNU and BCNU in the *uvrA* strain, the *recA* strain, as well as a double-deficient strain of CC102. The mutation profile of the double-deficient strain was similar to that of the NER-deficient strain, suggesting that an NER protects *E. coli* from mutations but not recombination. In addition, cell death was more pronounced in the *uvrA, recA* double-deficient strain than in the single-deficient strains.

**Conclusion:**

These results suggest that the toxic lesions induced by CENU were repaired additively or synergistically by NER and recombination. In other words, lesions, such as ICLs, appear to be repaired by NER and recombination independently.

## Background

Although many kinds of alkylating agents have been traditionally used as anti-cancer therapeutic agents, numerous studies have reported that alkylating agents can cause tumors due to genotoxicity, and that the DNA repair systems are strongly involved in expression of anti-carcinogenic and genotoxic functions [1–4]. In many cases, alkylation such as methylation, ethylation, or chloroethylation, at the *O*^6^ site of guanine causes DNA lesions that induce cytotoxicity and genotoxicity. Chloroethylnitrosoureas (CENUs), such as nimustine (ACNU) and carmustine (BCNU), are typical chloroethylating agents that are employed in tumor chemotherapy for treating several kinds of tumors, including lymphomas, melanomas, small cell lung cancer, Hodgkin disease, and cerebromas [5, 6]. The blood-brain barrier is a major obstacle in therapies for brain tumors [7], and since CENUs can cross the blood-brain barrier, they are useful and important as chemotherapeutic agents against brain tumors [8]. CENUs are thought to cause chloroethylation at several DNA sites, and among them, *O*^6^-chloroethylguanine is considered to be the most influential lesion in causing cytotoxicity and genotoxicity [4]. Alkylation of the *O*^6^ site of guanine is well-known to cause a base substitution from GC to AT [9]. *O*^6^-Chloroethylguanine is unstable and immediately transforms into *N*1-*O*^6^-ethenoguanine via circularization of its molecule as an intermediate to form an interstrand cross-link (ICL) between guanine and cytosine [10]. ICLs strongly inhibit DNA replication [11], and these ICL lesions are considered to be responsible for the cytotoxic effects of CENUs [4, 5]. However, ICLs might trigger not only cytotoxicity, but also genotoxicity, leading to tumor formation as a side effect of the chemotherapeutic treatment [5, 11, 12]. The involvement of *O*^6^-alkylguanine-DNA alkyltransferase (AGT) in the repair of chloroethylating guanine has been widely reported [6, 13–15]. Recently, the mechanism of ICL-repair as well as the cytotoxic effects of ICLs has been slightly clarified [11]. Previously Wiencke and Wiemels reported that BCNU was weakly mutagenic in Ames test using *Salmonella typhymurium his*G46 and TA1535 that detect the base substitution from G to A [16]. However, the relationship between the genotoxic lesions and mutagenesis remains unclear, and its repair pathway remains to be fully elucidated. In this study, we examined the mutations induced by ACNU and BCNU in *E. coli* strains CC101 to CC111, which can be used to detect reverse mutations due to each episome from F’101 to F’106 and frameshift mutations due to each episome from F’107 to F’111 [17, 18]. The mutantion frequency increased only in the *E. coli* CC102 strain, in which a GC to AT mutation was detected. In an exception, BCNU was mutagenic in *E. coli* CC104 strain only at high dose. Frameshift mutations were not detected in assays using strains CC107 to CC111. To determine the pathway responsible for repairing the CENU-induced lesions, we examined the frequencies of mutations induced by CENUs in the *ada, ogt* (AGT)-deficient strain, *uvrA* (NER)-deficient strain, mismatch repair (MMR)-deficient strain, *recA* (recombination)-deficient strain, and *uvrA* and *recA* double-mutant strain of CC102. The frequencies of mutations induced by ACNU and BCNU were elevated in the *ada, ogt* strain, indicating that *O*^6^-chloroethylguanine was formed, then repaired in part by AGT. The pathway for ICL repair is complicated, and it might be related to recombination. A significant decrease in survival was observed in the double-deficient *uvrA, recA* strain, while the mutant frequency was similar to that of the NER single-deficient strain, suggesting that in *E. coli,* NER and AGT prevent GC to AT mutations, and that NER and recombination independently prevent cytotoxic effects.

## Methods

### Materials

**Fig. 1:**
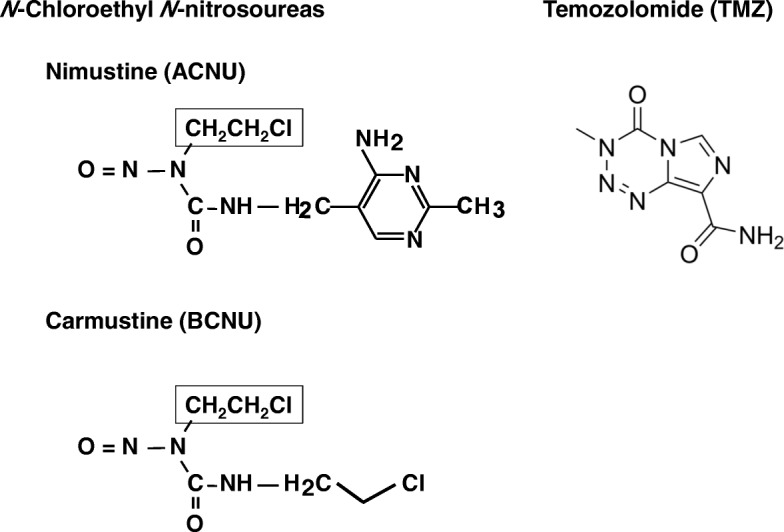
The structures of the mutagens used in this study

The ACNU (nimustine hydrochloride; 1-[(4-amino-2-methyl-5-pyrimidinyl)-.methyl]-3-(2-chloroethyl)-3-nitrosourea hydrochloride; CAS 55661–38-6) was purchased from Wako Pure Chemicals Co. (Osaka, Japan), and the BCNU (carmustine; 1,3-bis(2-chloroethyl)-1-nitrosourea; CAS 154–93-8) and temozolomide (TMZ; 3,4-dihydro-3-methyl-4-oxoimidazo-[5,1-*d*][1,2,3,5]tetrazine-8-carboxamide; CAS 85622–93-1) were purchased from Tokyo Chemical Industry Co., Ltd. (Tokyo, Japan). The structures of these compounds are shown in Fig. 1.

### Bacterial strains

*Escherichia coli* strains, each containing an F′ episome from CC101 to CC106, were used to detect base substitutions [17], and other *E. coli* strains, each containing an F′ episome from CC107 to CC111, were used to detect frameshift mutations [18]. The repair-deficient mutant strains with the CC102 episome used in this study are summarized in Table 1. All *E. coli* strains used in the mutagenesis experiments were derived from strain KA796 (*ara*, *thi* and Δ*pro- lac*) [19]. The wild-type strain (NR10832), the MMR-deficient derivatives (NR12896 (*mutS201::Tn5*), NR11102 (*mutL211::Tn5*), and NR12897 (*mutH471::Tn5*)), the NER-deficient derivative (NR12999 (*uvrA277::Tn10*)), and the recombination-deficient derivative (NR11312 (*recA56, srl::Tn10*)) have all been described by Negishi et al. [19]. The AGT-deficient mutant KT01121 (*ada-10*::Tn*10, ogt*::*cat*) has been reported by Taira et al. [20]. To lack the AGT activity completely, we use the double mutant in which both inducible gene (*ada*)-product (Ada) and constitutive gene (*ogt*)-product (Ogt) is deficient. Another NER-deficient strain, ZA2102 (*uvrA6, malE::Tn5*), was kindly gifted from Prof. T. Ohta (Tokyo University of Pharmacy and Life Sciences, Tokyo) [21]. A double mutant with *uvrA* and *recA,* SW102 (*uvrA6, malE::Tn5, recA56, srl*::Tn*10*), was constructed in this study using NR11312 as a host strain and ZA2102 as a donor strain, according to methods described previously [20, 22]. We confirmed that this strain was sensitive to ultraviolet (UV) irradiation and ENU due to a NER deficiency and a recombination deficiency, respectively.

**Table 1:** *E. coli* KA796 mutants used in this study and their spontaneous mutant frequencies

Strain^*a*^	Relevant genotype	Spontaneous mutant frequency^*b*^ (× 10^− 7^)	Ref.
NR12999	KA796, *uvrA277*::Tn*10*	0.24 ± 0.16	[18]
KT01121	KA796, *ada-10*::Tn*10, ogt*::*cat*	0.78 ± 0.27	[19]
NR12896	KA796, *mutS201*::Tn*5*	59.6 ± 11.4	[18]
NR11102	KA796, *mutL211*::Tn*5*	22.5 ± 9.63	[18]
NR12897	KA796, *mutH471*::Tn*5*	43.0 ± 16.5	[18]
NR11312	KA796, *recA56, srl*::Tn*10*	0.16 ± 0.1	[18]
ZA2102	*uvrA6, malE::Tn5*	< 0.1	[20]
SW102	*uvrA6, malE::Tn5, recA56, srl*::Tn*10*	0.1 ± 0.5	This study

*a:* All mutants contain CC102 F’episome

*b:* All spontaneous mutant frequencies shown in this table were measured in this study

### Bacterial mutation assay

**Fig. 2:**
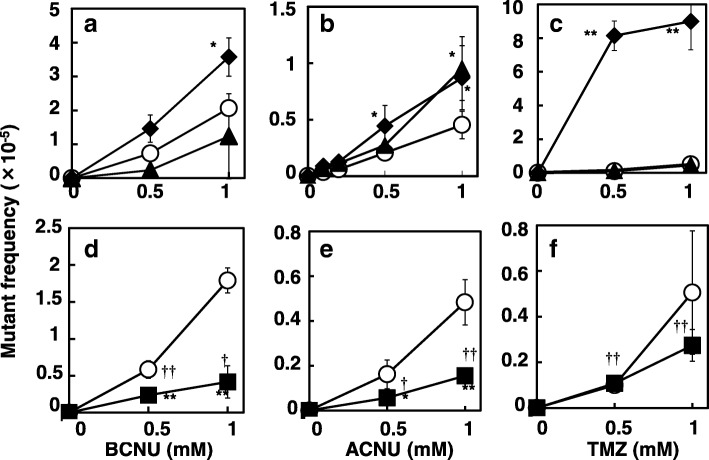
Mutant frequencies induced by BCNU (**a**, **d**), ACNU (**b**, **e**), and TMZ (**c**, **f**) in the wild-type (open circles), KT01121 (closed diamonds), NR12999 (closed triangles), and NR11312 (closed squares) strains. Statistical analysis was performed using the Student’s *t* test. **p <  0.01 and *p < 0.05 when compared to the mutation frequency of the wild-type strain. ††*p* < 0.01 and †p < 0.05 when compared to the mutant frequency of control without treatment

**Fig. 3:**
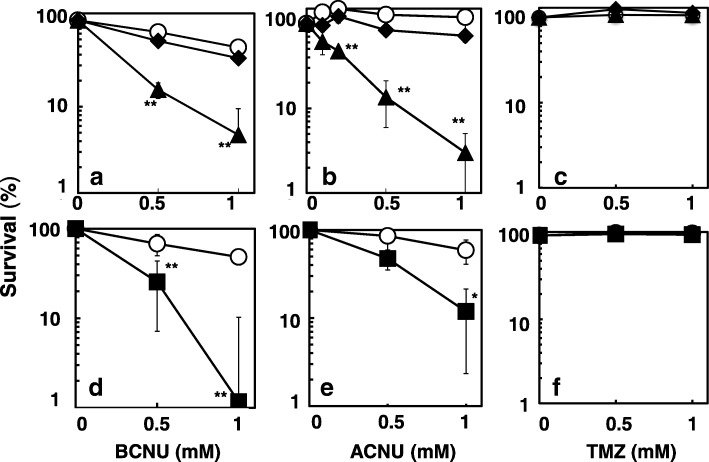
Cytotoxicity induced by BCNU (**a**, **d**), ACNU (**b**, **e**), and TMZ (**c**, **f**) in the wild-type (open circles), KT01121 (closed diamonds), NR12999 (closed triangles), and NR11312 (closed squares) strains. Statistical analysis was performed using the Student’s *t* test. **p < 0.01 and *p < 0.05 when compared to the mutant frequency of the wild-type strain

**Fig. 4:**
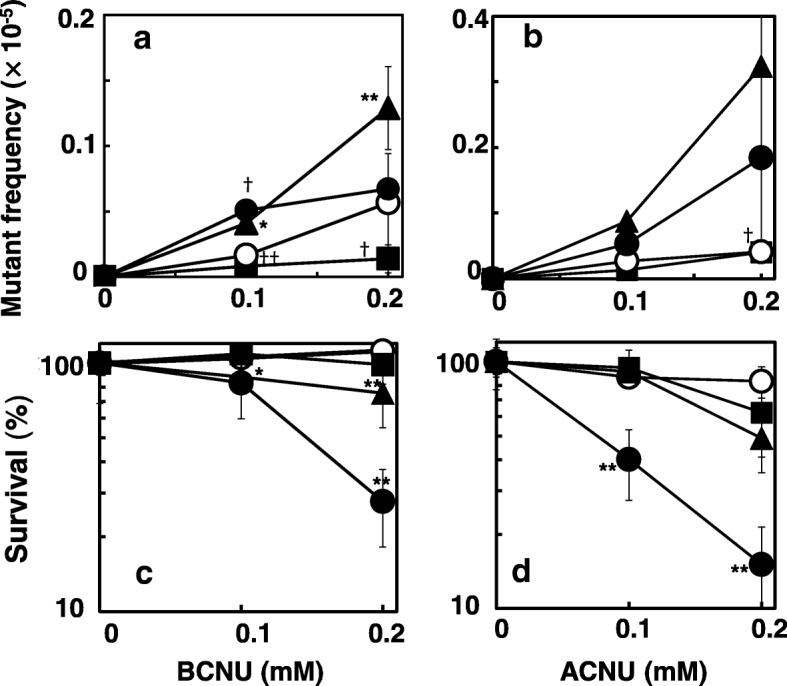
Frequency of mutations and cytotoxicity induced by BCNU (**a**, **c**) and ACNU (**b**, **d**) in the wild-type (open circles), NR12999 (closed triangles), NR11312 (closed squares), and SW102 (closed circles) strains. Statistical analysis was performed using the Student’s *t* test. **p < 0.01 and *p < 0.05 when compared to the mutation frequency of the wild-type strain. ††p < 0.01 and †p < 0.05 when compared to the mutant frequency of control without treatment

The *lac* allele of CC102 reverts to *lac*^+^ exclusively through a GC to AT transition [17]. We previously used this *lac* reversion system to detect the mutagenicity of alkylating agents in wild-type and mismatch-repair-deficient strains [20, 23, 24]. For mutagenesis, 0.1 ml of overnight cultures of each strain were incubated for 1 h at 37 °C with 0.5 ml of 0.1 M sodium phosphate buffer (pH 7.4) and 0.1 ml of mutagen solution dissolved in DMSO or water. We assayed in triplicate. Next, 0.1 ml of the treated cultures were spread onto minimal lactose plates to determine the number of revertants, and adequately diluted cultures were also spread onto minimal glucose plates to determine the total viable cell numbers. The doses of mutagens used in these assays were scarcely toxic. In almost all cases, the survival rates were greater than 80%, and in only a few cases did the survival rate decrease to approximately 60 to 70% at the highest dose used. The mutation frequencies were calculated by dividing the number of *lac*^+^ revertants by the number of total viable cells. In the experiment using double mutant (*uvrA* and *recA*), we performed the assay at lower dose of test compounds because ACNU and BCNU were more toxic in double mutant than in each single mutant. All experiments were independently repeated two or three times. Typical results are shown in Figs. 2, 3 and 4. Statistical analysis was performed using the Student’s *t* test.

## Results

### Mutation spectrum induced by ACNU and BCNU

Although the study of mutations induced by CENUs has mainly been performed in mammalian cells, in this study, we used an *E. coli* reversion assay to investigate the lesions resulted in CENU-induced mutations. First, we determined which base substitution or frameshift was induced by treatment with ACNU or BCNU using a series of *E. coli* strains, from CC101 to CC111. As shown in Table 2, the mutation frequency in CC102 increased significantly only after treatment with either compound, that is, a GC to AT transition occurred, whereas no other base substitution was detected, except for a GC to TA transversion resulting from BCNU treatment. No frameshift mutation was observed. In CC104, mutation was significantly detected with a higher dose of BCNU (5.19 ± 0.52 × 10^− 5^ at 1 mM, *p* <  0.01; compared to 0.45 ± 0.03 at 0 mM), suggesting that BCNU might also induce a GC to TA transversion. In the Ames test, ACNU and BCNU were mutagenic in TA100, 717 ± 194 revertants/ μmol and 1042 ± 122 revertants/μmol respectively, and ACNU was weakly, mutagenic in TA98 (92 ± 8.2 revertants/μmol). These results suggest that ACNU and BCNU induce a base substitution, that is, a GC to AT transition, and ACNU also induce frameshift mutation, that is, + 1(C/G).

**Table 2:** Mutant frequencies induced by ACNU and BCNU in *E. coli* series from CC101 to CC111

Strain	ACNU			BCNU		
	dose (mM)	MF (×10^−7^)	survival (%)	dose (mM)	MF (×10^−7^)	survival (%)
CC101	0	0.24 ± 0.42	100	0	0.24 ± 0.21	100
(AT to CG)	1	< 0.21	101.6 ± 6.5	0.5	0.72 ± 0.06	49.5 ± 4.2
CC102	0	0.42 ± 0.43	100	0	0.31 ± 0.02	100
(GC to AT)	1	33.0 ± 5.74**	102.1 ± 14.4	0.5	79.63 ± 23.6**	48.3 ± 10.5
CC103	0	< 0.17	100	0	< 0.11	100
(GC to CG)	1	< 0.18	94.1 ± 11.1	0.5	< 0.21	55.4 ± 3.9
CC104	0	0.31 ± 0.27	100	0	0.45 ± 0.03	100
(GC to TA)	1	0.62 ± 0.55	100.5 ± 12.8	0.5	1.75 ± 1.85	60.2 ± 13.3
CC105	0	0.64 ± 0.04	100	0	0.31 ± 0.53	100
(AT to TA)	1	< 0.12	151.0 ± 15.7	0.5	0.40 ± 0.35	63.6 ± 9.5
CC106	0	< 0.16	100	0	< 0.16	100
(AT to GC)	1	< 0.13	108.4 ± 13.6	0.5	< 0.22	57.6 ± 18.1
CC107	0	10.56 ± 1.86	100	0	6.90 ± 0.88	100
(+ 1 (G/C))	1	8.01 ± 1.60	107.6 ± 12.6	0.5	8.61 ± 1.54	65.8 ± 11.6
CC108	0	4.58 ± 1.17	100	0	3.39 ± 1.04	100
(+ 1 (G/C))	1	2.6 7± 0.70	111.9 ± 9.0	0.5	2.83 ± 2.18	87.1 ± 10.7
CC109	0	58. 37± 7.21	100	0	43. 88± 3.17	100
(+ 2 (CG/GC))	1	57.60 ± 12.7	109.2 ± 8.1	0.5	42.28 ± 13.38	91.4 ± 37.0
CC110	0	0.27 ± 0.46	100	0	0.12 ± 0.20	100
(+ 1 (A/T))	1	0.67 ± 0.30	141.2 ± 14.2	0.5	0.36 ± 0.32	75.3 ± 13.5
CC111	0	3.47 ± 0.18	100	0	6.85 ± 1.49	100
(−2 (A/T))	1	6.01 ± 2.21	67.4 ± 3.8	0.5	4.23 ± 0.76	90.6 ± 24.3

**; p < 0.01, significant increase from the corresponding control

### Effects of DNA repair deficiencies on the frequency of mutations induced by ACNU and BCNU

To determine which DNA lesions were induced by ACNU and BCNU, we examined the repair systems that can prevent the mutations induced by ACNU and BCNU using DNA repair-deficient mutants derived from CC102. An *ada, ogt* strain (KT01121) was used to examine the involvement of *O*^6^-chloroethylated guanine in the mutation, a NER-deficient strain (*uvrA*; MR12999) was used to examine not only chloroethyl but also more bulky adducts on the DNA, and a recombination-deficient strain (*recA*; NR11312) was used to examine ICLs in the DNA. As shown in Fig. 2a, BCNU was more mutagenic in the *ada, ogt* strain than in the wild-type strain, and the mutation frequency did not differ significantly between the *uvrA* strain and the wild-type strain. In contrast, ACNU was only slightly more mutagenic in the *ada, ogt* strain than in the wild-type strain, and the mutagenicity in the *ada, ogt* strain was similar to that in the *uvrA* strain (Fig. 2b). In addition, the mutagenicity of TMZ, a typical methylating anticancer drug, was markedly elevated in the *ada, ogt* strain (Fig. 2c). From these results, it was considered that the chloroethylated guanine at the *O*^6^ position of the DNA was partly repaired by AGT, as has been previously reported [4, 6]; however, AGT might be less efficient in repairing damage from chloroethylation than damage from methylation. In addition, ACNU might cause other DNA damage that is repaired by NER.

Chloroethylated *O*^6^-guanine sequentially forms ICLs via the circularization of ethenoguanine. It is thought that ICLs are repaired through recombination accompanied by NER. We examined the effects of recombination deficiency on the mutations induced by ACNU and BCNU using *recA* strain NR11312 (Fig. 2d and e) and the double-mutant *uvrA*, *recA* strain SW102 (Fig. 4a and b). Both compounds were very weakly but significantly mutagenic in NR11312, and the frequency of mutations decreased when compared to the wild-type strain. Recombination deficiency did not affect the mutagenicity and toxicity of TMZ (Fig. 2f). In *uvrA, recA* strain, the frequency of mutations induced by ACNU and BCNU appeared to be similar or slightly higher than that in the wild-type strain, and similar to that in the *recA* strain (Fig. 4a and b).

In summary, the mutation induced by ACNU and BCNU appears to be protected by AGT and NER whereas the efficacy of NER for BCNU-induced mutation appears to be lesser than that for ACNU. Recombination might assist in mutagenesis induced by ACNU and BCNU.

### Influences of DNA repair pathways on the cytotoxicity induced by ACNU and BCNU

ACNU and BCNU induce ICLs through *O*^6^-chloroethylguanine. The ICLs are believed to be the cytotoxic lesions and are proposed to be repaired by the combined action of NER and recombination [11]. Therefore we examined the cytotoxicity of ACNU, BCNU and TMZ using NER-deficient (*uvrA*), recombination-deficient (*recA*) *E. coli*. ACNU and BCNU caused strong cytotoxicity in the NER-deficient strain and *recA* strain (Fig. 3a, b, d and e), but TMZ was not toxic in any of the strains at the doses tested (Fig. 3c and f). These results show that NER and recombination remove ACNU- and BCNU-induced cytotoxic lesions. To examine the relationship between NER and recombination in the repair of the ACNU- and BCNU-induced cytotoxic lesions using *uvrA, recA* double mutant. The cytotoxic effects of ACNU and BCNU were much stronger in the *uvrA, recA* strain than in the *recA* or *uvrA* single-mutant strains (Fig. 4c and d). These results suggest that the lesions induced by CENUs might be repaired by NER and recombination independently.

### Effects of mismatch repair deficiencies on the frequency of mutations induced by ACNU and BCNU

We also examined whether MMR is involved in the repair of DNA lesions induced by CENUs, because it is well-known that MMR pathway involves in the processing alkylated guanine lesions by repair or cytotoxic effects [23–26]. Our results demonstrated that MMR was not responsible for the repair of chloroethylated lesions (Table 3). Thus, chloroethylated guanine does not appear to be a target for MMR.

**Table 3:** Mutant frequencies of ACNU and BCNU induced in MMR-deficient strains

		Mutant frequency (× 10^− 7^)					
	Doses (mM)	ACNU			BCNU		
		0	0.5	1	0	0.5	1
Strain	wild-type	0.39 ± 0.15	22.0 ± 5.83	39.2 ± 7.72	0.59 ± 0.62	45.2 ± 27.2	118 ± 77.3
	*mutS*	57.2 ± 9.11	71.8 ± 11.2	90.7 ± 9.15	49.6 ± 9.78	80.8 ± 6.43	158 ± 31.3
	*mutL*	15.5 ± 2.45	41.2 ± 2.60	96.4 ± 9.26*	24.1 ± 5.76	100 ± 38.2	215 ± 72.7
	*mutH*	34.8 ± 7.26	50.2 ± 9.05	78.9 ± 7.72	32.2 ± 4.09	107 ± 30.0	188 ± 75.9

These data are from 3 independent experiments with ACNU and from 2 independent experiments with BCNU

Statistical analysis was performed using the Student’s t test. *p < 0.05 compared with the mutant frequency for the wild-type

## Discussion

Chloroethylnitrosoureas (CENUs), such as ACNU and BCNU, are typical chloroethylating agents that are employed in tumor chemotherapy, especially for brain tumors due to their ability to pass through the blood-brain barrier [7]. CENUs cause the formation of *O*^6^-chloroethylguanine, followed by the formation of an ICL via *N*1-*O*^6^-ethenoguanine, and this ICL lesion blocks replication, leading to the cytotoxic effects of the CENUs [3–6]. However, these lesions are considered to be a double-edged sword, because they can also be genotoxic and mutagenic, and can lead to tumor formation. Unfortunately, the genotoxic mechanism of CENUs remains unclear. In this study, we examined the mutagenic effects induced by CENUs and the systems that repair the mutagenic DNA damage using an *E. coli* reversion assay system. This system can detect sequence-specific mutations easily. Previously, Tashima et al. reported that *recA*-lacking *E. coli* was very sensitive to BCNU, and that the lesions induced by BCNU are likely repaired by post-replication repair, that is, homologous recombination [27]; however, although they observed the cytotoxic effects of BCNU, they did not examine the mutations induced by BCNU. In our study, we first investigated the mutation spectrum caused by ACNU and BCNU using *E. coli* strains CC101 to CC111. The results showed that both chemicals induced GC to AT transition mutations in the CC102 strain, suggesting that chloroethylation occurred at the *O*^6^ position of guanine, because it has been reported that *O*^6^-chloroethylguanine induces GC to AT transitions via mispairing (reviewed in 3). These results are corroborated by previous reports that the toxicity of alkylating agents increased in cells lacking AGT or *O*^6^-methylguanine-DNA methyltransferase (MGMT) [2–4]. These results are in agreement with our data showing mutagenicity in *S. typhymurium* TA100. The therapeutic efficiency of ACNU for tumors is dependent on the MGMT expression status in tumor cells [28]. ACNU-induced sister chromatid exchanges were efficiently protected in HeLa cells transduced with human MGMT [29]. Preuss et al. observed that the protective effect of MGMT against cytotoxicity was specific to certain agents, that is, MGMT showed stronger protection against the cytotoxic effects of ACNU than those of BCNU in HeLa cells [14]. In addition, Becker et al. reported that skin tumors induced by topically applied ACNU were protected from cytotoxicity when human MGMT was expressed in the mice skin [30]. We observed a significant increase in the frequency of mutations induced by BCNU and ACNU in *ada, ogt E. coli*; in the case of ACNU, the mutant frequency was similar to that in the *uvrA* strain, while the frequency of mutations induced by a methylating agent, TMZ, was markedly elevated in the *ada, ogt* strain (Fig. 2). These results are in agreement with previous reports that *O*^6^-methylguanine is efficiently repaired by AGT, which we also confirmed using a series of repair-deficient *E.coli* CC102 strains [20]. However, from the present results, it appears that chloroethylation might not be as efficiently repaired by AGT as methylation at the *O*^6^ position of guanine. Previously, Mazon et al. revealed that in *E. coli,* an alkyltransferase-like protein prevents the toxicity that is induced by slightly larger *O*^6^-alkylG adducts through the enhancement of NER and inhibition of futile MMR cycles [31, 32]. In the present study, the lesions induced by ACNU appeared to be partly repaired by NER, because the frequency of mutations induced by ACNU in the NER-deficient (*uvrA*) strain increased to a similar level so that in the *ada, ogt* strain (Fig. 2b). Numata et al. reported that some steps of NER are involved in repairing ACNU-induced DNA damage in ACNU-sensitive CHO cells [33]. In our results, the frequency of mutations induced by BCNU was not affected by NER deficiency at higher doses of BCNU, but it appeared to be affected at lower doses. This suggested that CENU-induced lesions might be repaired not only by AGT, but also by NER, and ACNU might cause the formation of some bulky adduct, which are mainly repaired by NER.

BCNU was mutagenic in *E. coli* CC104 strain only at high dose, that is, BCNU may cause a GC to TA transversion. He et al. reported the protective effects of *E. coli* formamidopyrimidine (Fapy)-DNA glycosylase and human 8-oxoguanine-DNA glycolsylase on BCNU-induced DNA damage and cell death [34]. These results show that BCNU might induce *N*7-chloroethylguanine in DNA followed by the formation of a Fapy residue that can induce the GC to TA transversion. *N*7-Chloroethylguanine might be formed more efficiently by the treatment with BCNU at high dose.

*O*^6^-Chloroethylguanine rapidly transforms into unstable circularized *N*1-*O*^6^-ethenoguanine, then forms ICLs (reviewed in 4, 6). This transformation might be responsible for the less effective repair of *O*^6^-chloroethylguanine by AGT because *O*^6^-chloroethylguanine should exist shortly on DNA [35]. ICLs are considered to be chemotherapeutic DNA damage, because they efficiently disrupt DNA replication such as induction of replication-mediated double strand breaks [36], leading to cell death [5, 6]. It is well documented that recombination is involved in the repair of ICLs [5]. As such, we investigated whether recombination deficiencies affect the mutations induced by CENUs. ACNU and BCNU were toxic in *recA* strain of *E. coli,* and were less mutagenic than in the wild-type strain. In contrast, the mutagenicity of TMZ, which theoretically does not cause ICLs, was not affected, and no toxicity was observed at the doses tested (Fig. 3). These results suggest that the recombination of DNA is involved in the repair of ICLs, and that ICLs induce cell death if they are not repaired, as has been previously reported [5, 6]. NER appears to be responsible for the repair of ICLs, because the incision of strands is necessary for initiating recombination. As for the mechanism of ICL repair in *E. coli*, the NER-recombination model is proposed [37], that is, both NER and recombination are essential for the repair of ICLs. In this study, we constructed a new strain that was deficient in both NER and recombination to examine the involvement of NER and recombination in the toxicity and mutagenic effects of ACNU and BCNU. Our results showed that the toxicity of both compounds was stronger in the double-mutant strain than in each of the single-mutant strains; however, the mutagenic effects were the similar to those in the wild-type strain and in the NER-deficient strain (Fig. 4). In other words, the toxicity appeared to be caused by the additive or synergistic effects of NER deficiency and recombination deficiency, and the mutations appeared to be mainly caused by NER deficiency. These results suggest that the ICLs induced by CENUs are independently repaired by NER and recombination. If ICLs are repaired through the NER-recombination model, the toxicity should be similar between the double-mutant strain and each single-mutant strain, because both repair systems are epistatic to each other. Previously, Berardini et al. showed another pathway for the repair of ICLs induced by nitrogen mustard, that is, the NER/DNA polymerase II pathway, which is different from the NER/recombination pathway in *E. coli* [38]. Recently, Cole et al. reported that an incision by NER was not required for the repair of psoralen-induced ICLs in *E. coli,* and that *E. coli* may lack an efficient repair mechanism for damage, such as ICLs [39]. At replication forks, release of ICL probably causes DNA double strand breaks inducing cytotoxicity. As the repair of double strand breaks is mediated by RecA, replication-coupled repair of ICL might perform without aid of NER. On the other hand ICLs in non-replicated regions might be repaired by NER-mediated pathways such as the translesion pathway.

It is well documented that *O*^6^-methylguanine is efficiently repaired by both AGT (MGMT) and MMR repair systems [4]. Previously, we also revealed that methylating agents caused more mutations in AGT and MMR double-deficient strains than in each of the single-mutant strains [20]. In this study, we examined whether MMR was involved in the repair of chloroethylated lesions inducing reversion from GC to AT. As shown in Table 3, MMR does not appear to be responsible for the repair of chloroethylated lesions. There have been reports that the lesions induced by ACNU or BCNU might be repaired in a different manner from those induced by methylating agents, such as TMZ and dacarbazine, in mammalian cells [40, 41]. These data support our present finding that MMR does not appear to have a significant role in the repair of chloroethylated lesions.

## Conclusions

In conclusion, CENUs, such as ACNU and BCNU, induced GC to AT transitions in *E. coli* through the formation of *O*^6^-chloroethylguanine, and consequently, ICLs formed from the *O*^6^-chloroethylguanine via *N*1-*O*^6^-ethenoguanine, causing cytotoxicity. The formation of ICLs was not mutagenic, but toxic for *E. coli*. *O*^6^-Chloroethylguanine appears to be repaired by AGT, and perhaps also by NER, whereas ICLs appear to be independently repaired by recombination and NER. We summarize about repair pathway and potential DNA lesions in Table 4. As we used *E. coli* that enabled the detection of point mutations and frameshift mutations, there is a possibility that we could not observe the effects of recombination deficiencies on the mutations. In future studies, an *E. coli* strain for detecting mutations dependent on recombination should be used to examine changes in mutations due to recombination deficiencies.

**Table 4:** Summary

	ACNU		BCNU	
	Mutagenesis	Cytotoxicity	Mutagenesis	Cytotoxicity
Repair pathway	AGT NER	NER Recombination	AGT NER	NER Recombination
DNA lesions	*O*^6^-ChloroethylG *N*1-*O*^6^-ethenoG	ICL	*O*^6^-ChloroethylG *N*1-*O*^6^-ethenoG Fapy	ICL Fapy

## Abbreviations

ACNU: Nimustine (1-[(4-amino-2-methyl-5-pyrimidinyl)methyl]-3-(2-chloroethyl)-3-nitrosourea)
AGT: *O*^6^-alkylguanine-DNA alkyltransferase
BCNU: Carmustine (1,3-bis(2-chloroethyl)-1-nitrosourea)
CENU: Chloroethylnitrosourea
DMSO: Dimethylsulfoxide
ICL: Interstrand cross-link
MF: Mutant frequency
MMR: Mismatch repair
NER: Nucleotide excision repair
TMZ: Temozolomide (3,4-dihydro-3-methyl-4-oxoimidazo-[5,1-*d*][1,2,3,5]tetrazine-8-carboxamide)
